# Sine-SSA-BP Ship Trajectory Prediction Based on Chaotic Mapping Improved Sparrow Search Algorithm

**DOI:** 10.3390/s23020704

**Published:** 2023-01-08

**Authors:** Yuanzhou Zheng, Lei Li, Long Qian, Bosheng Cheng, Wenbo Hou, Yuan Zhuang

**Affiliations:** 1 School of Navigation, Wuhan University of Technology, Wuhan 430036, China; 2 Hubei Key Laboratory of Inland Shipping Technology, Wuhan University of Technology, Wuhan 430036, China

**Keywords:** ship trajectory prediction, sparrow search algorithm, sine chaos mapping, BP neural network

## Abstract

Objective: In this paper, we propose a Sine chaos mapping-based improved sparrow search algorithm (SSA) to optimize the BP neural network for trajectory prediction of inland river vessels because of the problems of poor accuracy and easy trapping in local optimum in BP neural networks. Method: First, a standard BP model is constructed based on the AIS data of ships in the Yangtze River section. A Sine-BP model is built using Sine chaos mapping to assign neural network weights and thresholds. Finally, a Sine-SSA-BP model is built using the sparrow search algorithm (SSA) to solve the optimal solutions of the neural network weights and thresholds. Result: The Sine-SSA-BP model effectively improves the initialized population of uniform distribution, and reduces the problem that population intelligence algorithms tend to be premature. Conclusions: The test results show that the Sine-SSA-BP neural network has higher prediction accuracy and better stability than conventional LSTM and SVM, especially in the prediction of corners, which is in good agreement with the real ship navigation trajectory.

## 1. Introduction

In recent years, the Yangtze River mainline has tried to meet the development of the economy, but because of the increasing flow of inland vessels and inland waterway congestion, ship traffic accidents have occurred frequently. Therefore, the use of the ship’s data to predict the ship’s trajectory has become a research hotspot for scholars at home and abroad. The ship’s data is mainly provided by the automatic identification system (AIS), which includes primarily maritime mobile service identification (MMSI), the ship’s latitude and longitude, course to ground, speed to ground, the ship’s draft, and so on. Currently, a series of trajectory prediction models have been proposed by domestic and foreign scholars. Li W G [1] used the state values of the grids to calculate the transfer probabilities between all neighboring grids to construct the original Markov model, and then combined the Markov model with an improved Bayesian approach, which has good performance with a small number of historical trajectories. Wesley Mathew [2] applied Hidden Markov to future location prediction, and also obtained good prediction results. Wiest J [3] modeled this with Gaussian mixed probabilities, which has the advantage that the results are not only predictions of individual trajectory points, but also of the entire distribution of future trajectories. Wang Q [4] proposed a dynamic trajectory prediction method combining dynamic measurement theory and gray system theory, and the accuracy of the final simulation results had only about one-fifth of its error relative to the Kalman filter. The literature [5,6,7,8] applied recurrent neural networks (LSTM, GRU) to ship trajectory prediction, and the final results are still quite satisfactory, but the prediction accuracy needs to be improved. In the literature [9,10,11], BP (back propagation) was used to predict ship tracks, but the accuracy of the prediction was reduced compared to LSTM. The reason for this is that the data input layer of the BP network is unordered, and the ship track waypoints are time-ordered, which is not reflected in this model, and the low accuracy of the single model has yet to be overcome. Qian L [12] proposed a genetic algorithm based on improved LSTM for inland ship trajectory prediction, which overcomes the low accuracy of a single model and improves the accuracy compared to the traditional LSTM. In order to improve the accuracy of the BP neural network in ship trajectory and prediction, as well as the fit of real ship navigation trajectory, this paper proposes a sparrow search algorithm SSA optimized BP neural network, based on Sine chaotic mapping improvement, for trajectory prediction of inland river ships (referred to as Sine-SSA-BP). Considering that BP neural networks are easily limited to local optima, making it difficult to improve prediction accuracy significantly, the weights and thresholds of BP neural networks are optimized using the Sparrow Search Algorithm (SSA). In addition, the initial population quality of the Sparrow Search Algorithm (SSA) is improved by using Sine chaos mapping in order to improve the ease of falling into local optimum solutions. The inputs to the algorithm are the longitude and latitude, speed, and heading of the ship, with the following moment of ship longitude and latitude as the target output. The experimental results show that the Sine-SSA-BP model is more accurate in predicting the ship’s navigational trajectory and better fits the ship’s navigational trajectory than the traditional BP neural network and SSA-BP models.

## 2. Trajectory Prediction Model

At present, the automatic identification system (AIS) of ships is one of the only effective ways to share information about ships for the outside world and to obtain real-time ship navigation data. To determine the specific navigation status information of ships is the data cornerstone of the development of future water intelligent transportation services; it can provide active identification for the outside world, track the ship navigation status, and ensure the safety and efficiency of the waterway [13]. In practice, AIS data can be influenced by many factors, so the input of experimental data requires the selection of relevant ship track point data to maintain a complete ship track route. The main navigational states of a ship in actual navigation include the ship’s latitude and longitude, speed, heading, and other data. The latitude and longitude represent the coordinate points of the ship’s position, and the speed and heading to the ground are the ship’s speed and heading relative to the ground, all of which have a significant impact on the prediction of the ship’s position. The ship’s position, speed to the ground and heading to the ground at t moment in time are used as input data to the model, and then the ship’s position (longitude, latitude) at the next moment in time is used as the output of the model. The latitude and longitude ($lat_{t}$,$lon_{t}$), the speed to the ground (sog), and the heading to the ground (cog) are selected as input data for the model, along with the task of predicting the future latitude and longitude of the ship. The input $y_{t}$ for the model at time t is given in the following equation.

(1) $$y_{t}=\left\{lat_{t},lon_{t},sog_{t},cog_{t}\right\}$$

Typically, the ship’s position at the next moment is determined by a series of temporal events, using the latitude and longitude of the future moment as the output of the model, representing a series of past temporal events as input. For the output $Y_{t+1}$ of the model at time t+1, see the following equation.

(2) $$Y_{t+1}=\left\{lat_{t+1},lon_{t+1}\right\}$$

Therefore, the prediction of a ship’s trajectory is a matter of using the time series of the ship’s historical state data to predict the ship’s position at the next moment. Ship trajectories are mainly characterized by non-linearity, randomness, trends, and periodicity. The ship trajectory belongs to a longer time series, where the previous moment is related to the future moment, and it is necessary to analyze the overall time series rather than the information of a particular moment. Therefore, the ship’s trajectory navigation point characteristics $y_{t-2},y_{t-1},y_{t}$ for three consecutive moments are used as input to the model, and the trajectory data $Y_{t+1}$ for t+1 moments are used as output.

## 3. Theoretical Approach

### 3.1. BP Neural Network

BP (Back Propagation) neural network has become one of the most effective neural networks applied in many fields. BP neural network is a back propagation learning algorithm of a multilayer network, i.e., forward propagation of input data and back propagation of error. This network can obtain mathematical mapping reflecting the inherent laws of the data, and strong nonlinear fitting ability, so that the error between the output value and the real value is minimized, and the effect of approximating various nonlinear continuous functions is achieved.

**Figure 1:**
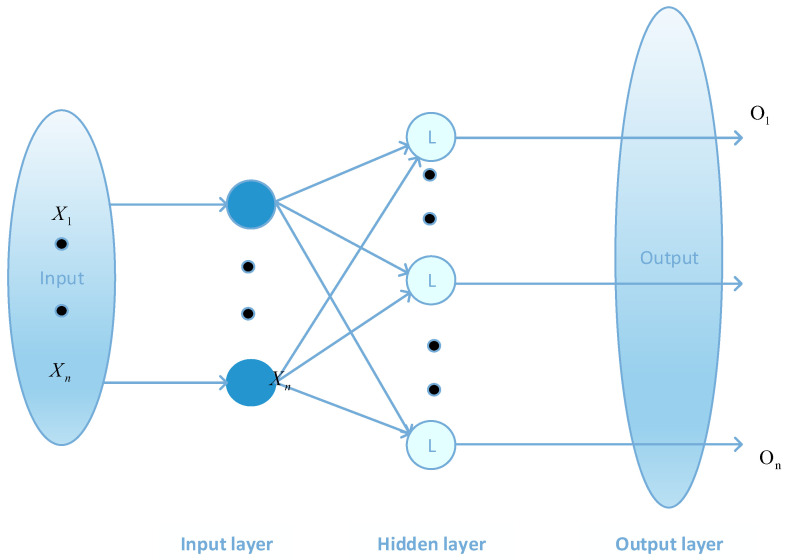
BP neural network structure diagram.

The BP neural network can be divided into three parts: the input layer, hidden layer, and output layer. The hidden layer consists of a multilayer network with full connectivity between layers, but there is no interconnection between units of the same layer. The network model of the BP neural network can realize multiple inputs and multiple outputs. A typical BP neural network structure is shown in Figure 1, which is a BP neural network with a hidden layer.

The relationship between the input and output of a neural network can be expressed by the following equation.

(3) $$o_{n}=\sum_{j=1}^{l}w_{jn}*\sigma \left(\sum_{i=1}^{k}w_{ij}x_{j}-\theta_{j}\right)$$

where l is the number of nodes in the hidden layer, k is the number of nodes in the input layer, and σ is the activation function.

Hornik [14] proved that “with only one hidden layer containing a sufficient number of neurons, a multilayer feedforward network can approximate a continuous function of arbitrary complexity with arbitrary accuracy.” For the determination of the number of hidden layer nodes, the setting range of the hidden layer neurons of a general multi-hidden layer BP neural network model is valued by an empirical formula [15], which is used as an initial value to be trialed by experiment.

(4) $$l=\sqrt{\left(m+n\right)}+a$$

where l is the number of nodes in the hidden layer, m is the number of nodes in the input layer, n is the number of nodes in the output layer, and a is generally taken as an integer between 1 and 10. In practice, the optimal range needs to be determined according to the empirical formula, and the optimal number of hidden layer neuron nodes is determined by the trial method.

The fitting process of a neural network consists of two processes: input of data and feedback of error. From the direction of data propagation, data propagation is a forward propagation process in the BP neural network, and network model error is backward propagation in the BP neural network. During the process of training the weights and thresholds of the network model, if the network model error is within the required accuracy and meets expectations, the learning process can be stopped. Conversely, not meeting the expectations means that the network model is under-trained, and the weights and thresholds need to be adjusted until they are within the required accuracy. The mathematical expression is as follows.

The mathematical expression of forward propagation of input data.

(5) $$\{\begin{array}{c} net_{j}=\sum_{i=0}^{n}u_{ij}x_{j},(j=1,2,\ldots ,m)\\ v_{j}=f(net_{j}),(j=1,2,\ldots ,m) \end{array}$$

(6) $$\{\begin{array}{c} net_{k}=\sum_{j=0}^{n}u_{jk}x_{j},(k=1,2,\ldots ,L)\\ v_{j}=f(net_{k}),(j=1,2,\ldots ,L) \end{array}$$

Equation (5) represents data from the input layer to the hidden layer, and Equation (6) represents data from the hidden layer to the output layer.

The mathematical expression for back propagation of data errors is:

(7) $$\{\begin{array}{l} E^{q}=\frac{1}{L}\sum_{k=1}^{L}{\left(d_{k}^{q}-y_{k}^{q}\right)}^{2}\\ E_{1}=\frac{1}{L}\sum_{k=1}^{L}{\left(d_{k}-f(\sum_{j=0}^{m}w_{jk}v_{j})\right)}^{2}\\ E_{2}=\frac{1}{L}\sum_{k=1}^{L}(d_{k}-f({\sum_{j=0}^{m}w_{jk}f(\sum_{i=0}^{n}u_{ij}x_{i})))}^{2} \end{array}$$

In Equation (7), $E^{q}$ denotes the mean square error, $E_{1}$ denotes the middle layer error propagation, and $E_{2}$ denotes the input layer error propagation.

From the above equations, it is clear that the error changes with the adjustment of the weights by the constant adjustment of the weight vectors $w_{jk}$ and $u_{ij}$ between the layers. Therefore, in order to improve the learning rate, the error can be continuously reduced by adjusting the weights only.

### 3.2. Sparrow Search Algorithm (SSA)

The Sparrow search algorithm is a new population intelligence optimization algorithm proposed by XUE J [16]. Compared with other population intelligence algorithms, SSA is a new population intelligence optimization algorithm superior to Gray Wolf Optimization Algorithm (GWO), Particle Swarm Optimization (PSO), Gravity Search Algorithm (GSA), and other algorithms. The central idea of the population intelligence optimization algorithm is to search for the optimal solution of the solution space distributed in a certain range by simulating the movement and behavior laws of some things or organisms in nature [17], and the principle of bionics is as follows.

Sparrow population’s foraging can be abstracted into two types of behavior: predation and anti-predation. Predatory behavior is composed of producers–scroungers: producers have more resources and determine the search direction of the population, and scroungers follow the producers and move to guide the population to search and forage. At the same time, scroungers increase their own predation rate, and some scroungers monitor the producer to facilitate food competition or foraging around it. The identities of producers and scroungers change with iteration, with scroungers having more resources becoming producers, but the overall ratio of both to the population remains the same. Anti-predation behavior consists of early-warning agents, which alert the entire population when it is threatened by a predator or when it is aware of the danger. When the warning value is greater than the safe value, the producer leads the population to migrate to other feasible, safe areas, and sparrows at the edge of the population move faster to safe areas, while sparrows in the middle area of the population walk randomly to follow the population. The mathematical model is as follows:

Producer Location Updates:

(8) $$X_{i,j}^{t+1}=\{\begin{array}{l} X_{i,j}^{t}\cdot \exp(\frac{-i}{\alpha \cdot T}),if\;R_{2}<ST\\ X_{i,j}^{t}+Q\cdot L,if\;R_{2}\geq ST \end{array}$$

where $X_{i,j}^{t+1}$ denotes the position of the ith sparrow in the jth dimension at the tth iteration, T denotes the maximum number of iterations, Q is a random number obeying the standard normal distribution, α is a uniform random number in the range of (0, 1], and L denotes a matrix of 1*d, and each element of the matrix is 1. $R_{2}$ is the warning value; if the warning value is reached, then the sparrow population has encountered danger and needs to take measures ($R_{2}\in [0,1]$). ST is the safety value, i.e., if the sparrow population is within the safety value, the sparrow population can move normally (ST∈[0.5,1]).

Scrounger Location Updates:

(9) $$X_{i,j}^{t+1}=\{\begin{array}{l} Q\cdot \exp(\frac{X_{worst}^{t}-X_{i,j}^{t}}{i^{2}}),if\;i>n/2\\ X_{P}^{t+1}+|X_{i,j}^{t}|\cdot A^{+}\cdot L,othwewise \end{array}$$

where $X_{P}^{t+1}$ denotes the position of the best producer at the t+1th iteration, $X_{worst}^{t}$ denotes the global worst position at the tth iteration, Q is a random number obeying the standard normal distribution, L denotes a 1*d matrix and each element of the matrix is 1. A is a 1*d matrix and each element of the matrix is randomly assigned −1 or 1, and $A^{+}=A^{T}{\left(AA^{T}\right)}^{-1}$.

Early-warning Agent Location Updates:

(10) $$X_{best}^{t+1}=\{\begin{array}{l} X_{best}^{t}+\beta \cdot |X_{i,j}^{t}-X_{best}^{t}|,if\;f_{i}>f_{g}\\ X_{i,j}^{t}+K\cdot (\frac{\left|X_{i,j}^{t}-X_{worse}^{t}\right|}{f_{i}-f_{w}+\varepsilon }),if\;f_{i}=f_{g} \end{array}$$

where $X_{best}$ is the current global optimal position and β is a random number obeying a normal distribution. K∈(−1,1), denotes the sparrow movement direction and step control parameters. $f_{i}$ is the current individual sparrow adaptation value. $f_{g}$ is the current global optimal adaptation value, and $f_{w}$ is the current global worst adaptation value. ε is an infinitesimal constant to avoid a zero denominator.

### 3.3. Chaotic Mapping to Optimize Initial Populations

Chaotic mappings are used to generate chaotic sequences, which are sequences of randomness generated by simple deterministic systems. Generally, chaos is characterized by nonlinearity, ergodicity, randomness, overall stability, and local instability. In the field of optimization, chaotic mappings can be used as an alternative to pseudo-random number generators to generate chaotic numbers between 0 and 1. It has been experimentally demonstrated that using chaotic sequences for population initialization, selection, crossover, and mutation affects the whole algorithm process, and often achieves better results than pseudo-random numbers [18,19].

Currently, the existing chaotic mappings can be divided into two categories according to the dimensionality: one-dimensional chaotic mappings and high-dimensional chaotic mappings, while one-dimensional chaotic mappings, with simple structure and fast computation, generate chaotic sequences faster compared to high-dimensional chaotic mappings [20]. The paper mainly focuses on one-dimensional chaotic mapping as a study.

#### 3.3.1. Mathematical Expressions for Sine Mapping

The Sine mapping is obtained from the deformation of the sine function, and is widely used in major fields due to its simple structure. Its mathematical expression is:

(11) $$x_{i+1}=S(x_{i})=\mu \cdot \sin(\pi x_{i})$$

where the control parameter μ takes values in the range [0, 1] and the initial value $x_{0}$ takes values in the range (0, 1).

#### 3.3.2. Mathematical Expressions for Logistic Mapping

The one-dimensional logistic mapping is a very simple chaotic mapping from the mathematical form, this system has extremely complex dynamical behavior and has a wide range of applications in the field of confidential communications. the mathematical expression of which is as follows.

(12) $$x_{i+1}=\mu \cdot x_{i}(1-x_{i})$$

where $x_{0}\notin (0,0.25,0.5,0.75,1.0),\mu \in [0,4],x_{i}\in (0,1)$.

#### 3.3.3. Mathematical Expressions for Tent Mapping

The tent mapping is a function with parameter β and is a segmented linear mapping. the mathematical expression of which is as follows.

(13) $$x_{i+1}=\{\begin{array}{l} x_{i}/\beta ,x_{i}\in (0,\beta ]\\ \left(1-x_{i})/(1-\beta ),x_{i}\in (\beta ,1\right] \end{array}$$

where β∈(0,1).

**Figure 2:**
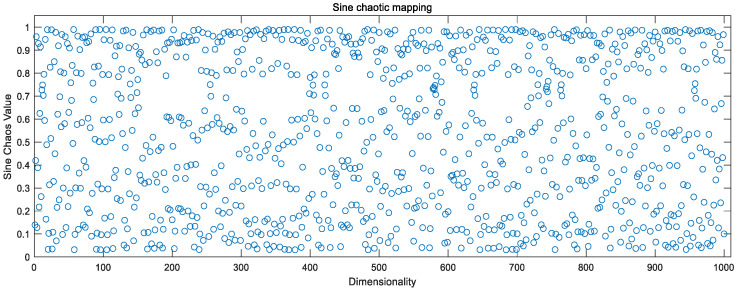
Sine chaotic mapping values.

**Figure 3:**
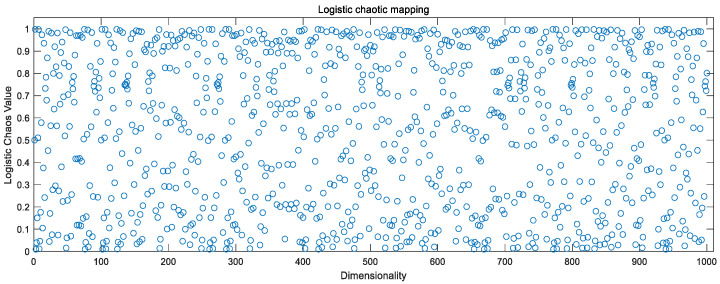
Logistic chaotic mapping values.

**Figure 4:**
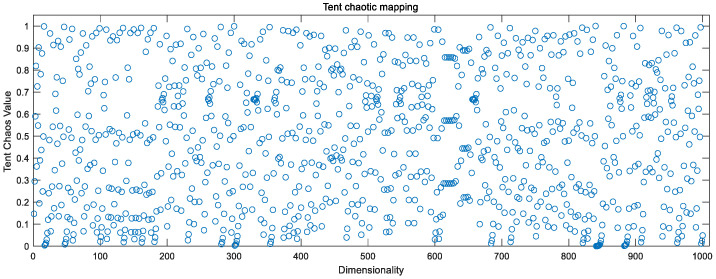
Tent chaotic mapping values.

Combining Figure 2, Figure 3 and Figure 4, the above chaotic mapping, given the initial values and control parameters, runs with the system, i.e., iteratively.

### 3.4. Improved Sparrow Search Algorithm Based on Chaotic Mapping

**Figure 5:**
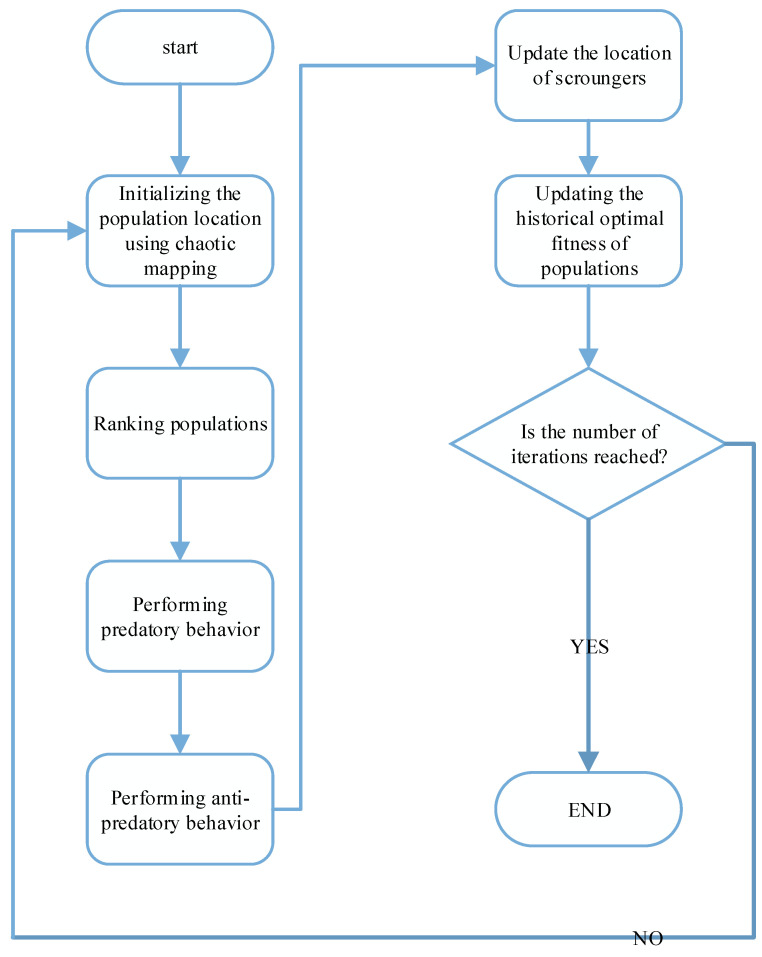
Chaotic mapping algorithm flowchart.

Applying chaos mapping to SSA increases the uniformity of the initial solution distribution, improves the efficiency and traversal uniformity of the optimal search, improves the population search capability, and, to some extent, overcomes the shortcomings of the population intelligence algorithm, such as the reduction of population diversity when approaching the optimal solution, the tendency to fall into local optimum, and the reduction of search accuracy. The flow chart is shown in Figure 5. The algorithm flow is as follows:

- Step 1 Initialize the sparrow population according to the chaotic mapping of Equations (11)–(13), set the population size N, iteration number T, warning value PD, predator SD, scroungers proportion, and warning proportion.
- Step 2 Calculate individual fitness in the sparrow population, rank all sparrow individual fitnesses, and find the global optimal fitness $f_{g}$ and the global worst fitness $f_{w}$.
- Step 3 Update the producer location information according to Equation (8).
- Step 4 Update the scrounger location information according to Equation (9).
- Step 5 Update the early-warning agents’ position information according to Equation (10).
- Step 6 Calculate the sparrow population fitness and reordering to update the sparrow population location.
- Step 7 Judge whether the algorithm reaches the maximum number of iterations or meets the requirements of solution accuracy. If it meets the requirements, the loop ends and the objective function is output; otherwise, return to step 2 until the end condition is met.

### 3.5. Fitness Function

The fitness function can define the optimal individual in the population. Based on the different characteristics of individuals, the fitness function is used to determine the fitness values of different individuals. The choice of the fitness function directly affects the optimization performance of the algorithm, which, in turn, affects the prediction performance. In this paper, we construct the fitness function based on the mean square error between the predicted and true values of ship navigation position coordinates. Its individual fitness function is defined as:

(14) $$fitness=\frac{1}{n}\sum_{t=1}^{n}{\left(y_{t}-p_{t}\right)}^{2}$$

where n denotes the sample size, $y_{t}$ denotes the true value, and $p_{t}$ denotes the predicted value.

## 4. Sine-SSA-BP Model

**Figure 6:**
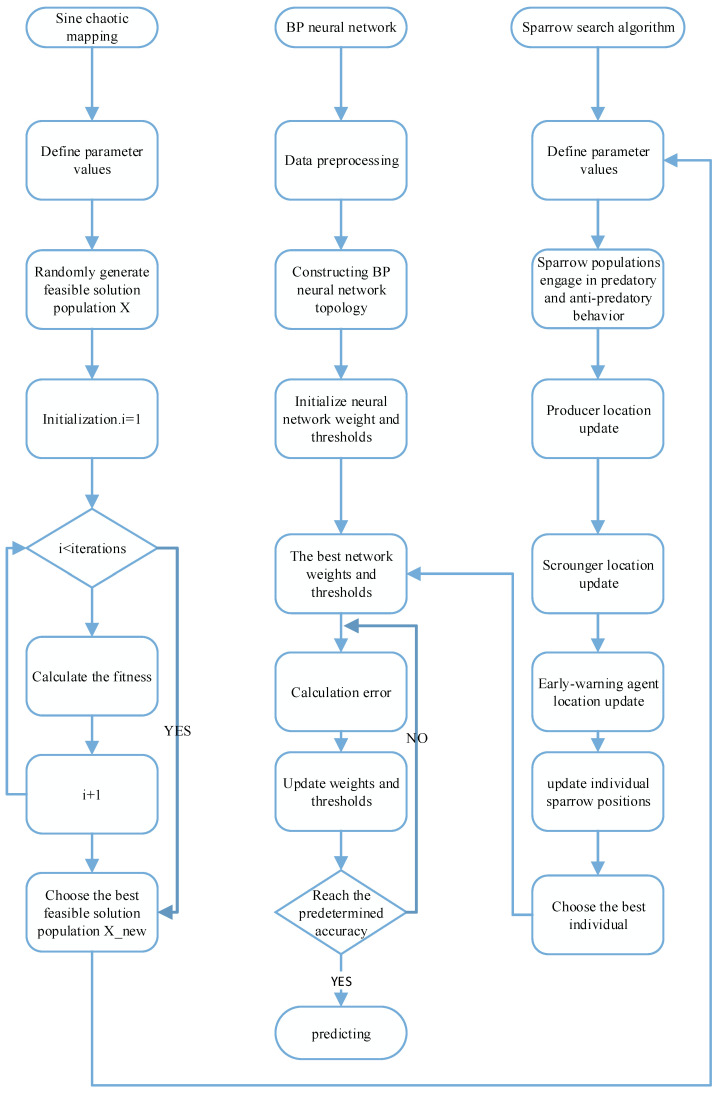
Sine-SSA-BP model framework diagram.

The sparrow optimization algorithm (SSA) introduces sine chaos mapping to increase the diversity of populations, improve the search performance and pioneering performance of the algorithm, and to avoid falling into local optimum. The flow chart is shown in Figure 6. The steps of the Sine-SSA-BP model are as follows:

- Step 1 Build a standard BP neural network, Sine-SSA initialization, including population size N, number of producers $PD_{NUM}$, number of sparrows for reconnaissance warning $SD_{NUM}$, dimension D of the objective function, upper and lower bounds lb, ub of initial values, the maximum number of iterations T, or solution accuracy ε.
- Step 2 Input data and pre-processing.
- Step 3 Determine the topology of the neural network.
- Step 4 Determine the weights $w_{i}$, thresholds $b_{i}$, and nodes dim (D = dim) of the BP neural network.
- Step 5 Apply the Sine chaos mapping in Section 3.4 to initialize the population and generate an N of D-dimensional vectors $Z_{i}$.
- Step 6 Calculate the fitness $f_{i}$ of each sparrow, select the current optimal fitness $f_{g}$ and its corresponding position xb, and the current worst fitness $f_{w}$ and its corresponding position xw.
- Step 7 Select the top $PD_{NUM}$ sparrows with the best adaptation as producers and the remaining as scroungers, and update the positions of producers and scroungers according to Equations (8) and (9).
- Step 8 Randomly select $SD_{NUM}$ sparrows from the sparrow population for reconnaissance warning, and update their positions according to Equation (10).
- Step 9 Recalculate the fitness value $f_{i}$ for each sparrow after one iteration is completed.
- Step 10 Based on the current state of the sparrow population, update the optimal position xb and its fitness $f_{g}$ experienced by the whole population, and the worst position xw and its fitness $f_{w}$.
- Step 11 Determine whether the algorithm runs to the maximum number of iterations or the solution accuracy. If yes, the loop ends and the result of the optimization search is output; otherwise, it returns to Step 7.
- Step 12 Output the optimal weight $w_{i}$ and threshold $b_{i}$.

## 5. Experiment Design

### 5.1. Experiment Environment

The experiment is run on a personal PC with 16 G of running memory and an AMD Ryzen 5 5600X CPU, a PC based on Windows operating system, and MATLAB 2019b software. The experimental data were selected from 21,867 data from three ships in the Nanjing-Chongqing section of the Yangtze River. The MMSI were 413**23, 413**89, and 413**98. A total of 5423 sets of data from ship 413**23 were used as the experimental data, and the training and test sets were divided according to 80% and 20%, with the first 4338 sets as the training set and the last 1085 sets as the test set. The training set is input to the network for training. The 10,000 sets of data from ship 413**89 and 6444 sets of data from ship 413**98 are used to verify the generalization ability of the model.

After several experiments, the number of intermediate layer nodes in the Sine-SSA-BP model was adjusted to 6, and the learning rate was 0.001. The number of chaotic Sine mappings was 91, and the number of populations was 90; the number of initial populations in SSA was 30, and the number of iterations was 50, which a producer ratio of 0.3 and a scrounger ratio of 0.7.

### 5.2. Experimental Data and Preprocessing

**Figure 7:**
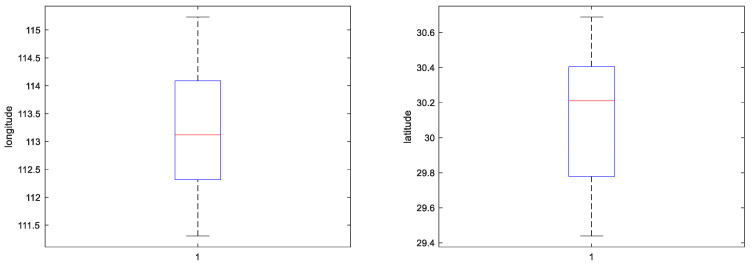
Distribution of longitude and latitude.

**Figure 8:**
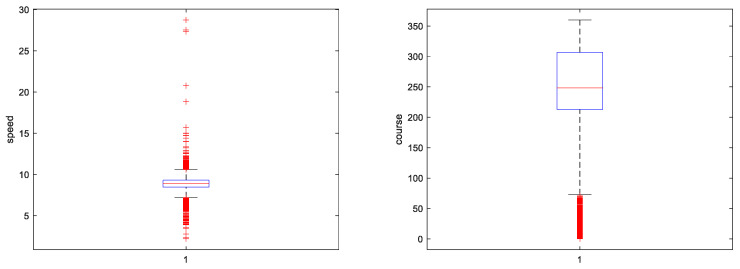
Distribution of speed and course.

The experimental data in this paper is the data collection carried out by the subject team several times. The data in this paper is mainly concentrated in the middle and lower reaches of the Yangtze River. The AIS data can be incomplete due to various factors, such as the missing navigation status information on the time period, while the AIS data can also have time series redundancy. Therefore, in the data pre-processing process, it is necessary to retain the original sample of the ship trajectory, as well as to add and remove data. The preprocessing process of the AIS data collected in this paper is as follows [21]:

1. MMSI is not a 9-bit data value.
2. AIS attribute information contains a large amount of data with null values.
3. In this paper, the longitude range of the track point is set to [111.3051,115.2309], the latitude range is set to [29.4386,30.6876], the speed range is set to [2.218,28.738], the course range is set to [0–360], and the distribution of research data after AIS data cleaning is shown in Figure 7 and Figure 8.
4. Treatment of missing values.
5. Data normalization can prevent the weight of other data in the total data, due to the large difference in individual data values. Therefore, Min–Max Normalization is used to map each data between [0,1] to ensure that all data are treated fairly by the neural network, and the Min–Max Normalization transformation is shown in Equation (15):

(15) $$X_{m}=\frac{X-X_{\min}}{X_{\max}-X_{\min}}$$

where: X is the original value, $X_{\min}$ and $X_{\max}$ are the minimum and maximum values, respectively, and $X_{m}$ are the normalized data.

### 5.3. Evaluation Metrics

In the model for error analysis, since the error calculation is trying to know the size of the deviation of the true point from the predicted point, the *MSE* (Mean Square Error) is an excellent measure of the error between the true situation and the predicted result. The *RMSE* (Root Mean Square Error) also reflects the offset between the true and predicted points, and is a commonly used accuracy evaluation metric [22]. The units of latitude and longitude used for the evaluation metrics are all (°). The mathematical expressions of *MSE* and *RMSE* are shown in Equations (16) and (17), and the smaller the two evaluation metrics indicates better model performance. In addition, since the loss function of the BP network is *MSE*, to make the article more rigorous, *MAE* (Mean Absolute Error) and *MAPE* (Mean Absolute Percentage Error) are introduced to verify each other. The mathematical expressions of *MAE* and *MAPE* are shown in Equations (18) and (19).

(16) $$MSE=\frac{1}{n}\sum_{t=1}^{n}{\left(y_{t}-p_{t}\right)}^{2}$$

(17) $$RMSE=\sqrt{\frac{1}{n}\sum_{t=1}^{n}{\left(y_{t}-p_{t}\right)}^{2}}$$

(18) $$MAE=\frac{1}{n}\sum_{t=1}^{n}\left|y_{t}-p_{t}\right|$$

(19) $$MAPE=\frac{1}{n}\sum_{t=1}^{n}\frac{\left|y_{t}-p_{t}\right|}{y_{t}}$$

where n denotes the number of samples, $y_{t}$ denotes the true value, and $p_{t}$ denotes the predicted value.

## 6. Results and Analysis

### 6.1. Comparison of Prediction Accuracy of SSA-BP Models with Different Structures

**Figure 9:**
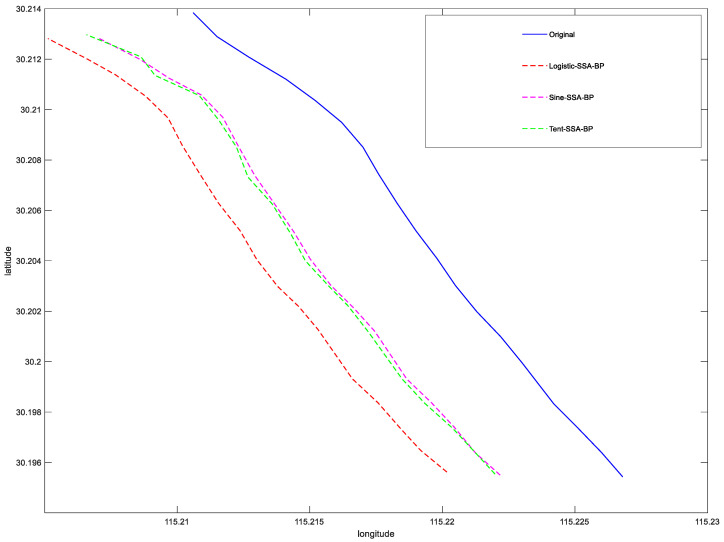
Comparison of trajectory prediction of three different structural models.

In order to determine the enhancement of the predictive power by chaotic mapping and sparrow search algorithm (SSA), the prediction accuracy of Tent+ SSA-BP, Logistic +SSA-BP, and Sine -SSA-BP models were compared. The overall mean square error (*MSE*) and root mean square error (*RMSE*) of the three models, latitude *MSE*, latitude *RMSE*, longitude *MSE*, and longitude *RMSE*, are shown in Table 1. It can be seen in the Table 1 and Figure 9 that: the latitude and longitude errors of Sine-SSA-BP are all below Tent+ SSA-BP and Logistic + SSA-BP, representing that the chaotic mapping Sine for SSA- BP has a better prediction enhancement effect, which indicates the stability and good prediction ability of the model Sine-SSA-BP.

### 6.2. Different Models Predict Visualization

**Figure 10:**
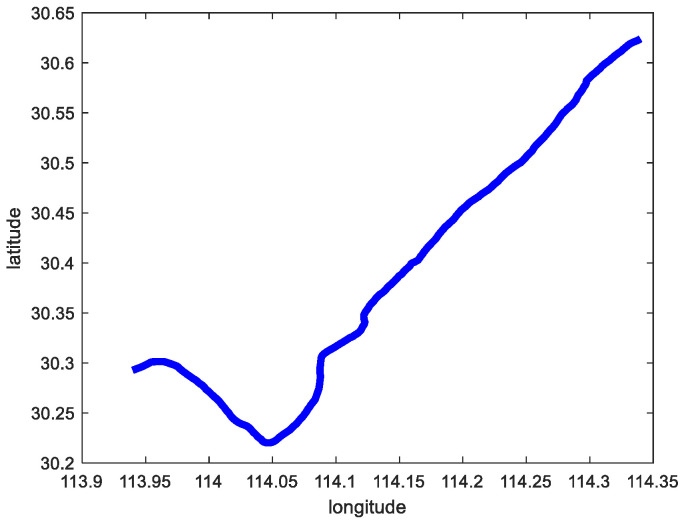
The original trajectory of scenario 1.

**Figure 11:**
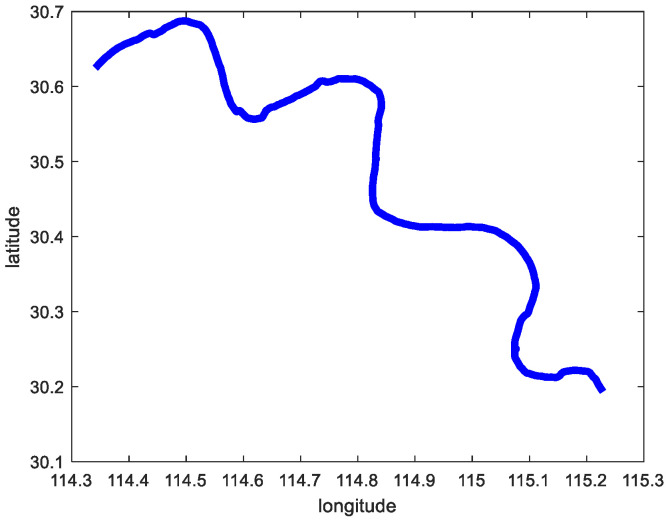
The original trajectory of scenario 2.

To verify the error between the real and predicted trajectories of the Sine-SSA-BP model, the corresponding parameters were input during the model training and prediction, and different parameters had different effects on the model training and prediction [23]. The model prediction ability is verified by adjusting the parameters to the model and using the test set to test the prediction ability of the model [24]. In this paper, two scenarios are selected for the test set. Figure 10 shows the original trajectory of scenario 1, and Figure 11 shows the original trajectory of scenario 2.

#### 6.2.1. Scenario 1 Visualization Comparison Analysis

**Figure 12:**
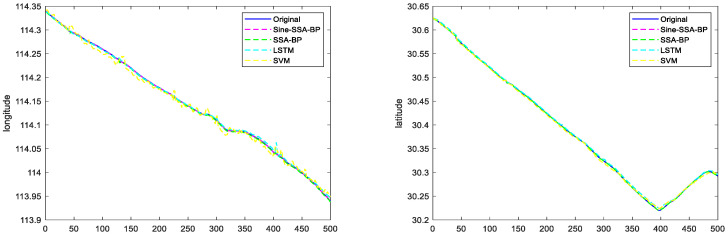
Plots of predicted longitude and latitude for different model trajectories in Scenario 1.

**Figure 13:**
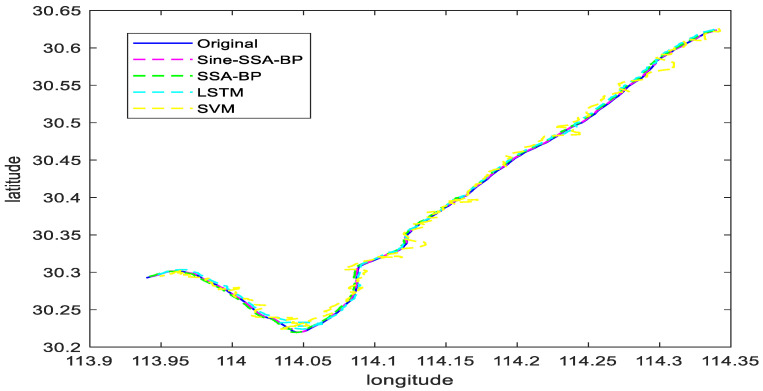
Comparison of trajectory prediction of different models in Scenario 1.

Scenario 1 contains the trajectory of the ship sailing in a straight line and a small angle corner. The ship navigation data (latitude, longitude, speed, and heading to the ground) of scenario 1 are used as the input signals of the network. The latitude and longitude of the ship’s position are determined as the output signals of the network, and the experimental results are shown in Figure 12. The comparison of trajectory prediction of different models in Scenario 1 is shown in Figure 13.

To further prove the feasibility and effectiveness of the text method, the Sine-SSA-BP, SSA-BP, SVM, and LSTM models are compared and analyzed, and the experimental results are shown in Figure 13, which shows that:

(1) For the trajectory prediction in scene 1, the SVM model performs the worst, i.e., the ship trajectory predicted in a straight line is substantially the same, but the position information has a large deviation, which is due to the weak generalization ability of SVM, which makes it easy to fall into local extremes.

(2) The SSA-BP model and LSTM model have comparable prediction effects, but in terms of their overall effects, the SSA-BP model is slightly better than the LSTM model. This is because the network hyperparameters of the LSTM model are difficult to take the optimal solution manually, which makes the prediction performance lower than that of the SSA-BP model.

(3) The Sine-SSA-BP model performs the best and can predict the ship’s sailing position more accurately. The reason is that the population intelligence algorithm tends to be premature, while the Sine chaotic mapping can improve the initial population and reduce the premature problem of the population intelligence algorithm.

#### 6.2.2. Scenario 2 Visualization Comparison Analysis

**Figure 14:**
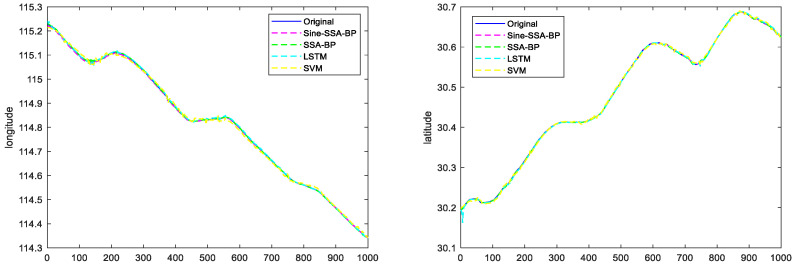
Plots of predicted longitude and latitude for different model trajectories in Scenario 2.

**Figure 15:**
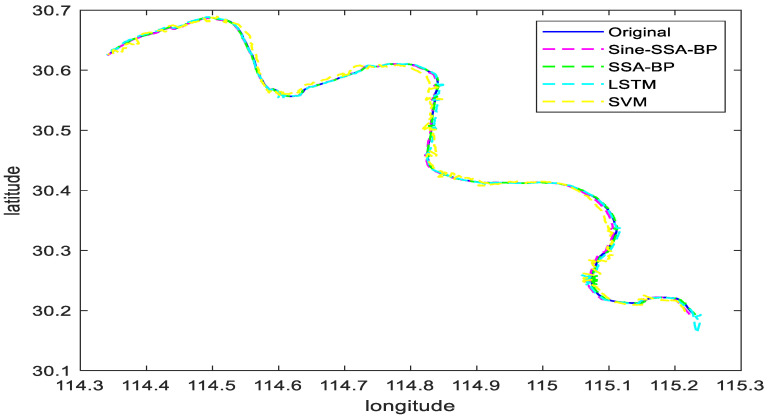
Comparison of trajectory prediction of different models in Scenario 2.

Scenario 2 contains the ship trajectory of the ship under continuous corners. From Figure 11, it can be seen that scenario 2 has the characteristics of continuous corners. The longitude and latitude of this trajectory are predicted by using Sine-SSA-bp, SSA-BP, SVM, and LSTM models, and the experiments are shown in Figure 14. From Figure 14, it can be seen that the ship longitude and latitude predicted by the method in this paper basically keep overlapping with the actual ship navigation longitude and latitude. Similarly, the trajectory is predicted by the above methods separately, and the experimental results are shown in Figure 15. As can be seen from Figure 15, even for ships with large turning amplitudes and continuous turns, the trajectory prediction performance of the method proposed in this paper is higher, and the effect is the best, and the sailing position of the ship can be predicted effectively, which further proves the effectiveness and feasibility of the method in this paper.

Combining Figure 12, Figure 13, Figure 14 and Figure 15, it can be seen that the latitude accuracy of different mode predictions is better than the longitude accuracy because the span of latitude is smaller than the span of longitude.

### 6.3. Analysis of Metrics of Different Models

To verify the prediction accuracy of the Sine-SSA-BP model, SVM, LSTM, SSA-BP, and Sine-SSA-BP are compared in this paper for experiments. The error comparison of the four models is shown in Table 2, which compares the mean square error and root mean square error of SVM, LSTM, SSA-BP, and Sine-SSA-BP models in overall, latitude, and longitude, and it can be seen that the *MSE*, *RMSE*, latitude *MSE*, latitude *RMSE*, longitude *MSE*, longitude *RMSE*, latitude *MAE*, latitude *MAPE*, longitude *MAE*, and longitude *MAPE* are lower, which indicates that the model can simulate the ship navigation state in the trajectory prediction. The addition of the sparrow search algorithm corrects the reduction of significant errors generated on the prediction results, and the Sine-SSA-BP model has the best prediction performance.

The prediction trajectories of the four models in small-angle corners are not as good as the straight-line trajectory prediction. The prediction trajectories of the model Sine-SSA-BP in the straight-line and corners are most consistent with the real trajectories. The prediction errors are smaller, especially in the small-angle corners prediction, because the input data before and after changes a lot. Sine-SSA-BP can make the input data close to the future moment data with the smallest error, so it is better in the sudden change. Sine-SSA-BP can make the input data close to the future moment data with minimum error, so it is better in processing the data, and has high accuracy for small angle corners prediction. The predicted trajectories of SVM in small-angle corners are deviate farther from the actual trajectories, and the errors in the prediction of corners are larger than the original trajectory values. Sine-SSA-BP is better than SVM, LSTM, and SSA-BP in straight line and small angle corners prediction. Table 3 shows the comparison of prediction errors in Scenario 1, and Table 4 shows the comparison of errors in Scenario 2.

The performance of model LSTM and model SSA-BP are not as good as that of model Sine-SSA-BP in continuous corners, where model SVM starts to show a more significant deviation, and the predicted trajectory deviates from the true trajectory. In contrast, model SSA-BP and model LSTM show increased error in continuous corners, but the predicted trajectory matches the true trajectory. It can be seen from Figure 15 and Table 4 that model Sine-SSA-BP outperforms the latter three models in predicting the trajectory of continuous corners, and the trajectory fits better. The *MSE, RMSE, MAE*, and *MAPE* are smaller. The time-series data of the historical and future moments of Sine-SSA-BP input also predicted the continuous abrupt data for seven successive changes in the continuous corners. They thus had a better agreement on the continuous curve prediction. In summary, the Sine-SSA-BP model performs better than model SVM, LSTM, and model SSA-BP in a straight line, small angle bend, and continuous bend, and the predicted trajectories have some reference value.

### 6.4. Sine-SSA-BP Model Generalization Validation

To verify the generalization ability of the model Sine-SSA-BP, on the basis of the original data of 5423 groups of ships 413**23, and then 10,000 groups of ships 413**89 and 6444 groups of ships 413**98, the generalization ability of SVM, LSTM, SSA-BP, and Sine-SSA-BP models are compared in Table 5. The *MSE, RMSE, MAE,* and *MAPE* of the model Sine-SSA-BP is the smallest compared with SVM, LSTM model, and SSA-BP in the prediction of ship data of 10,000 groups and 6444 groups, which indicates that the model has a certain generalization ability to predict the trajectory of the incoming and outgoing ships.

## 7. Conclusions

The hybrid model Sine-SSA-BP is proposed in this paper, which combines the Sine chaos mapping to improve the population quality of SSA and the optimization of SSA to BP neural network, and establishes the hybrid model Sine-SSA-BP. The model can accurately predict the ship trajectory in straight lines, small-angle corners, and continuous corners, and improves the prediction ability of the model in multiple scenarios. Compared with the traditional LSTM and SVM models, the prediction accuracy in latitude and longitude is significantly improved, and the predicted trajectories in small-angle corners and continuous corners are more consistent and have higher stability, which will play a positive role in maintaining the traffic of waterways and the safe navigation of ships. Therefore, the method proposed in this paper can meet the demand in certain scenarios in terms of real-time and has good generalization.

Ship performance and spatial characteristics should be fully considered in future research on ship trajectory prediction, and the influence of ship performance and spatial characteristics on ship trajectory prediction should be explored. The prediction accuracy of the hybrid model proposed in this paper needs further improvement, and the next step can be to study the application of a neural network model based on the ship model in the ship trajectory prediction research.

## Figures and Tables

**Table 1:** Comparison of SSA-BP models of different structures.

Errors	Logistic + SSA-BP	Sine + SSA-BP	Tent + SSA-BP
*MSE*	$7.717\times 10^{-6}$	$7.543\times 10^{-7}$	$2.213\times 10^{-6}$
*RMSE*	$1.310\times 10^{-3}$	$8.685\times 10^{-4}$	$1.488\times 10^{-3}$
*MSE*(longitude)	$3.308\times 10^{-6}$	$1.428\times 10^{-6}$	$4.325\times 10^{-6}$
*RMSE*(longitude)	$1.819\times 10^{-3}$	$1.195\times 10^{-3}$	$2.780\times 10^{-3}$
*MSE*(latitude)	$1.253\times 10^{-7}$	$8.041\times 10^{-8}$	$1.008\times 10^{-7}$
*RMSE*(latitude)	$3.540\times 10^{-4}$	$2.836\times 10^{-4}$	$3.174\times 10^{-4}$
*MAE*(longitude)	$1.319\times 10^{-3}$	$9.346\times 10^{-4}$	$1.450\times 10^{-3}$
*MAPE*(longitude)	$1.148\times 10^{-5}$	$8.171\times 10^{-6}$	$1.261\times 10^{-5}$
*MAE*(latitude)	$2.330\times 10^{-4}$	$1.705\times 10^{-4}$	$1.996\times 10^{-4}$
*MAPE*(latitude)	$7.649\times 10^{-6}$	$5.600\times 10^{-6}$	$6.556\times 10^{-6}$

**Table 2:** Comparison of different model errors.

Errors	LSTM	SVM	SSA-BP	Sine-SSA-BP
*MSE*	$7.7906\times 10^{-6}$	$3.2329\times 10^{-5}$	$2.7708\times 10^{-6}$	$1.3990\times 10^{-6}$
*RMSE*	$2.7912\times 10^{-3}$	$5.6859\times 10^{-3}$	$1.6646\times 10^{-3}$	$1.1828\times 10^{-3}$
*MSE*(longitude)	$9.8655\times 10^{-6}$	$5.6593\times 10^{-5}$	$5.3433\times 10^{-6}$	$2.6912\times 10^{-6}$
*RMSE*(longitude)	$3.1409\times 10^{-3}$	$7.5228\times 10^{-3}$	$2.3116\times 10^{-3}$	$1.6405\times 10^{-3}$
*MSE*(latitude)	$5.7156\times 10^{-6}$	$8.0658\times 10^{-6}$	$1.9833\times 10^{-7}$	$1.0679\times 10^{-7}$
*RMSE*(latitude)	$2.3907\times 10^{-3}$	$2.8400\times 10^{-3}$	$4.4535\times 10^{-4}$	$3.2679\times 10^{-4}$
*MAE*(longitude)	$2.3770\times 10^{-3}$	$6.5305\times 10^{-3}$	$1.6188\times 10^{-3}$	$1.3776\times 10^{-3}$
*MAPE*(longitude)	$2.0728\times 10^{-5}$	$5.6964\times 10^{-5}$	$1.4086\times 10^{-5}$	$1.2025\times 10^{-5}$
*MAE*(latitude)	$1.6865\times 10^{-3}$	$2.4382\times 10^{-3}$	$3.2454\times 10^{-4}$	$2.1432\times 10^{-4}$
*MAPE*(latitude)	$5.5457\times 10^{-5}$	$8.0112\times 10^{-5}$	$1.0645\times 10^{-5}$	$7.0431\times 10^{-6}$

**Table 3:** Comparison of trajectory prediction errors in Scenario 1.

Errors	LSTM	SVM	SSA-BP	Sine-SSA-BP
*MSE*	$7.2572\times 10^{-6}$	$2.6353\times 10^{-5}$	$1.6558\times 10^{-6}$	$2.0230\times 10^{-7}$
*RMSE*	$2.6939\times 10^{-3}$	$5.1336\times 10^{-3}$	$1.2868\times 10^{-3}$	$4.4978\times 10^{-4}$
*MAE*	$2.1866\times 10^{-3}$	$3.9472\times 10^{-3}$	$9.5750\times 10^{-4}$	$3.5015\times 10^{-4}$
*MAPE*	$4.8733\times 10^{-5}$	$6.1663\times 10^{-5}$	$1.0241\times 10^{-5}$	$6.6560\times 10^{-6}$

**Table 4:** Comparison of trajectory prediction errors in Scenario 2.

Errors	LSTM	SVM	SSA-BP	Sine-SSA-BP
*MSE*	$8.0572\times 10^{-6}$	$3.5317\times 10^{-5}$	$4.0551\times 10^{-6}$	$1.2706\times 10^{-6}$
*RMSE*	$2.8385\times 10^{-3}$	$5.9428\times 10^{-3}$	$2.0137\times 10^{-3}$	$1.1272\times 10^{-3}$
*MAE*	$1.9543\times 10^{-3}$	$4.7529\times 10^{-3}$	$1.2824\times 10^{-3}$	$7.1519\times 10^{-4}$
*MAPE*	$3.2773\times 10^{-5}$	$7.1975\times 10^{-5}$	$1.5220\times 10^{-5}$	$9.1808\times 10^{-6}$

**Table 5:** Model generalization ability.

Errors	LSTM	SVM	SSA-BP	Sine-SSA-BP
*MSE*(10,000)	$9.0857\times 10^{-4}$	$1.5904\times 10^{-3}$	$1.9600\times 10^{-5}$	$1.9600\times 10^{-5}$
*RMSE*(10,000)	$3.0142\times 10^{-2}$	$3.9879\times 10^{-2}$	$4.4272\times 10^{-3}$	$4.4272\times 10^{-3}$
*MSE*(6444)	$1.9602\times 10^{-4}$	$1.0467\times 10^{-3}$	$3.1066\times 10^{-5}$	$3.1066\times 10^{-5}$
*RMSE*(6444)	$1.4001\times 10^{-2}$	$3.2352\times 10^{-2}$	$5.5737\times 10^{-3}$	$5.5737\times 10^{-3}$
*MAE*(10,000)	$1.7117\times 10^{-2}$	$3.6335\times 10^{-2}$	$3.4079\times 10^{-3}$	$3.4079\times 10^{-3}$
*MAPE*(10,000)	$1.5128\times 10^{-4}$	$3.2037\times 10^{-4}$	$3.0058\times 10^{-5}$	$3.0058\times 10^{-5}$
*MAE*(6444)	$6.5692\times 10^{-3}$	$2.7724\times 10^{-2}$	$5.3029\times 10^{-3}$	$5.3029\times 10^{-3}$
*MAPE*(6444)	$6.1419\times 10^{-5}$	$2.5925\times 10^{-4}$	$4.9617\times 10^{-5}$	$4.9617\times 10^{-5}$

## Data Availability

Not applicable.
